# Multifactor Effects and Evidence of Potential Interaction between *Complement Factor H* Y402H and *LOC387715* A69S in Age-Related Macular Degeneration

**DOI:** 10.1371/journal.pone.0003833

**Published:** 2008-12-02

**Authors:** Sanna P. Seitsonen, Päivi Onkamo, Gang Peng, Momiao Xiong, Petri V. Tommila, Päivi H. Ranta, Juha M. Holopainen, Jukka A. Moilanen, Tapani Palosaari, Kai Kaarniranta, Seppo Meri, Ilkka R. Immonen, Irma E. Järvelä

**Affiliations:** 1 Department of Ophthalmology, University of Helsinki, Helsinki, Finland; 2 Department of Medical Genetics, Haartman Institute, University of Helsinki, Helsinki, Finland; 3 Department of Biological and Environmental Sciences, University of Helsinki, Helsinki, Finland; 4 School of Life Science, Fudan University, Fudan, China; 5 Human Genetics Center, University of Texas School of Public Health, Houston, Texas, United States of America; 6 Department of Ophthalmology, University of Oulu, Helsinki, Finland; 7 Department of Ophthalmology, University of Kuopio, Kuopio, Finland; 8 Department of Bacteriology and Immunology, Haartman Institute, University of Helsinki, Helsinki, Finland; 9 Laboratory of Molecular Genetics, Helsinki University Central Hospital, Helsinki, Finland; Peninsula Medical School, United Kingdom

## Abstract

**Background:**

Variants in the *complement cascade genes and the LOC387715/HTRA1,* have been widely reported to associate with age-related macular degeneration (AMD), the most common cause of visual impairment in industrialized countries.

**Methods/Principal Findings:**

We investigated the association between the *LOC387715* A69S and complement component *C3* R102G risk alleles in the Finnish case-control material and found a significant association with both variants (OR 2.98, *p* = 3.75×10^−9^; non-AMD controls and OR 2.79, *p* = 2.78×10^−19^, blood donor controls and OR 1.83, *p* = 0.008; non-AMD controls and OR 1.39, *p* = 0.039; blood donor controls), respectively. Previously, we have shown a strong association between *complement factor H (CFH)* Y402H and AMD in the Finnish population. A carrier of at least one risk allele in each of the three susceptibility loci (*LOC387715*, *C3, CFH*) had an 18-fold risk of AMD when compared to a non-carrier homozygote in all three loci. A tentative gene-gene interaction between the two major AMD-associated loci, *LOC387715* and *CFH*, was found in this study using a multiplicative (logistic regression) model, a synergy index (departure-from-additivity model) and the mutual information method (MI), suggesting that a common causative pathway may exist for these genes. Smoking (ever vs. never) exerted an extra risk for AMD, but somewhat surprisingly, only in connection with other factors such as sex and the *C3* genotype. Population attributable risks (PAR) for the *CFH*, *LOC387715* and *C3* variants were 58.2%, 51.4% and 5.8%, respectively, the summary PAR for the three variants being 65.4%.

**Conclusions/Significance:**

Evidence for gene-gene interaction between two major AMD associated loci *CFH and LOC387715* was obtained using three methods, logistic regression, a synergy index and the mutual information (MI) index.

## Introduction

Age-related macular degeneration (AMD [MIM 603075]) is the most common cause of irreversible visual loss in the elderly in the Western world. It is characterized by drusen deposits in its early forms. The late forms are geographic atrophy and exudative AMD, which especially affects central vision [1]. AMD is a complex disease with both genetic susceptibility and environmental risk factors contributing to the disease pathogenesis. Of the environmental risk factors, age and smoking have most consistently been identified as major risks [2], [3]. In the past few years, research into the genetics of AMD has been very successful. An association between the Y402H polymorphism of the *complement factor H* (CFH [MIM 134370]) gene on chromosome 1 and AMD has been confirmed in several Caucasian populations [4]–[19]. In addition, an association between the *LOC387715/high temperature requirement factor A-1 (HTRA1)* locus on chromosome 10q and AMD both in Caucasian, and in Japanese and Chinese populations has been found [11], [20]–[31]. Due to the close proximity of *LOC387715/HTRA1* and controversial results in functional studies [27], [32]–[34] the true disease-associated variant at this locus has remained so far unknown.

Most recently, a common polymorphism (rs2230199) in the *complement component 3* (C3 [MIM 120700]) gene of complement cascade was associated with AMD in two case-control sets [35] and confirmed in a large case-control sample [36]. With regard to the previously observed association to *CFH* this is an interesting observation because CFH is known to inhibit activation of C3 in the complement system [37].

We have previously shown that the *CFH* Y402H polymorphism is associated with AMD in Finnish patients [16]. Here we investigated whether the *LOC387715* A69S (rs10490924), *HTRA1* promoter (rs11200638), or *C3* R102G (rs2230199) variants are associated with AMD in the Finnish population. In addition, we compared three different statistical methods to estimate the combined risks and gene-gene and gene-environment interaction of CFH Y402H, LOC387715 A69S, C3 R102G, and smoking to the development of AMD.

## Materials and Methods

### Patient material

The patient material comprised 332 Finnish patients with AMD attending the Departments of Ophthalmology of Helsinki (n = 201), Oulu (n = 10) and Kuopio (n = 39) University Hospitals, or private offices and outpatient clinics (n = 82) [16]. A total of 151 patients were sporadic cases with no known relatives with AMD and 181 were familial cases with at least one first or second degree relative with AMD. Of the 154 sporadic AMD cases in our previous study [16] we excluded two patients because of genotyping failure and one of the sporadic cases was reported to have a novel AMD case in his family and was thus transferred to the familial group. Of the 181 previous familial cases one from Kuopio University Hospital could not be genotyped because of insufficient sample. Otherwise the patient material was the same as in our previous study (Table S1). We had two control groups, as previously reported [16]: 105 age-matched non-AMD controls with no large drusen and no or minimal focal pigmentary abnormalities and 350 anonymous blood donor controls.

In the family members of the index patients, the AMD stage was verified from fundus photographs or angiograms in 45 (60.0%), from the medical records in 28 (37.3%) and from an examination by a retinal specialist belonging to the study group in two of the patients (2.7%). In the rest of the subjects (index cases, sporadic cases and non-AMD controls) the AMD stage was verified from fundus photographs or angiograms in 338 (93.4%), from an examination by a retinal specialist belonging to the study group in four (1.1%) and from medical records in 20 (5.5%) of the subjects. The verification of the AMD status of patients was carried out as described in [16]. Blood samples for genotyping were obtained from all the patients with AMD and control individuals. We chose to use two control groups, 105 ophthalmologically investigated non-AMD controls, and 350 anonymous blood donor controls. Information on smoking was available in only the non-AMD controls. The study was approved by the Ethics Committee of the Helsinki University Eye and Ear Hospital and The Red Cross Blood Transfusion Service, Helsinki, Finland, and performed in accordance with the Declaration of Helsinki. Informed consent was obtained from all of the subjects after explanation of the nature and possible consequences of the study.

### Information on smoking

Information about the smoking history of the study participants was obtained by telephone. The following data were recorded: Had a participant ever smoked, the ages at which he/she started and quit (if he/she had quit) smoking, as well as the numbers of cigarettes smoked per day during that period. Then, the numbers of pack-years were calculated (cigarettes smoked per day×years smoked/20 [cigarettes per pack]). A binary variable, never/ever, was based on the information obtained on whether a study participant had smoked <one pack-year (never-smoker), or >one pack-year (ever-smoker) in his/her lifetime.

### Genotyping

The genotyping procedure for *CFH* was described in the previous article [16]. The DNA of the study subjects was amplified by the polymerase chain reaction (PCR) and sequenced using primers for *LOC387715* rs10490924 forward 5-GGTGGTTCCTGTGTCCTTCA-3 and reverse 5-GGGGTAAGGCCTGATCATCT-3 [29], for *HTRA1* rs11200638 forward 5-ATGCCACCCACAACAACTTT-3 and reverse 5-CGCGTCCTTCAAACTAATGG-3 [27], and for *C3* rs2230199 forward 5-GGAACAGACCCCTGACAATG-3 and reverse 5-CTTGTGGTTGACGGTGAAGA-3. Amplification was performed in a DNA 2720 Thermal Cycler (ABI, Foster city, CA, USA). The polymerase chain reaction conditions were as follows: 5 min at 94°C followed by 35 cycles of the denaturation step: 30 s at 94°C; the annealing step: 30 s at 59°C (rs10490924), 52°C (rs 11200638) and 60°C (rs2230199); the elongation step: 45 s at 72°C; and final extension for 7 min at 72°C terminated the reaction after final annealing. Sequencing was performed using cycle sequencing with the Big Dye Terminator kit (version 3.1) supplied by Applied Biosystems (ABI, Foster City, CA, USA), and reactions were run on an ABI 3730 capillary sequencer according to the manufacturer's instructions.

### Statistical analysis

Allele and genotype frequencies were estimated by direct counting. Deviations from Hardy-Weinberg Equilibrium (HWE) were tested with the standard Chi square test separately in cases and controls, to identify possible genotyping problems. No deviations were found (p>0.5 for all tests). The overall success rate in genotyping was 99.5%. The *LOC387715* rs10490924 and *HTRA1* rs11200638 were found to be in almost perfect LD with each other (D′ = 0.99).

### Individual SNPs

The allelic and genotypic associations of the individual loci (*LOC387715* rs10490924, *HTRA1* rs11200638, *C3* rs2230199) were measured by the standard Pearson's Chi square test with one degree of freedom (or Fisher's exact test, where necessary). Marginal odds ratios (OR) and their confidence intervals were estimated for all significantly associated loci to assess the strength of the association. This was carried out with R scripts freely available on the Internet (R Package Epitools (http://sites.google.com/site/medepi/epitools, function odds ratio). Furthermore, population attributable risks (PAR) were estimated with Levin's formula; (100%×proportion of exposed×(OR−1))/(proportion of exposed×(OR−1)+1)), where the proportion of exposed is the frequency the allele or genotype in the blood donor controls).

### Descriptive analyses of G×G interactions

Joint ORs for pairs of loci (*CFH* Y402H and *LOC387715 A69S*; *LOC387715 A69S* and *C3* rs2230199; *CFH* Y402H and *C3* rs2230199) were calculated for each 2-locus genotype separately, using the non-risk double homozygote genotype (TTGG, GGCC, TTCC, respectively) as a reference (Table 1). All patients with AMD (n = 332) were compared to blood donor controls (n = 350). The estimation was carried out with the aforementioned R package Epitools.

**Table 1:** Two-locus odds ratios (OR), 95% confidence intervals (95%CI), and *p*-values for different genotypic combinations of the *complement factor H* Y402H (genotypes *CC*/*CT*/*TT*) and the *LOC387715* A69S (genotypes *TT*/*TG*/*GG*) polymorphisms.

Genotypic combination of *CFH* and *LOC38771*5	OR	(95%CI)	*p*-value	Number of risk alleles
*TTGG*	1.00			0
*CTGG*	2.31	(1.09–5.26)	3.42×10^−2^	1
*CCGG*	8.83	(4.11–20.61)	3.52×10^−9^	2
*TTGT*	2.89	(1.20–7.31)	2.41×10^−2^	1
*CTGT*	8.48	(4.19–18.84)	1.18×10^−10^	2
*CCGT*	14.41	(6.69–33.85)	1.08×10^−13^	3
*TTTT*	21.38	(6.26–91.39)	2.08×10^−7^	2
*CTTT*	17.35	(7.03–46.92)	2.75×10^−11^	3
*CCTT*	26.57	(9.71–83.25)	1.66×10^−12^	4

Eight genotype combinations are compared to the non-risk genotype combination *TTGG*. The risk alleles are *C* (*CFH*) and *T* (*LOC387715*). All the patients with AMD (n = 332) are compared to the blood donor controls (n = 350). Note the strong correlation between number of risk alleles present in each two-locus genotype and the estimated odd's ratios.

### Multivariable and interaction analyses

Multivariable logistic regression was used for assessing the relationship between the independent variables and the outcome, estimating the effects of the individual SNPs and covariates (age, sex, smoking), and for dissecting potential gene-gene (G×G) and gene-environment (G×E) interactions in our data. This was carried out with the SPSS Binary logistic regression modeling procedure, in which stepwise backward variable selection procedure was used to screen out the informative covariates from the uninformative. We coded the loci genotypes following the notation presented by Cordell (2002) and North et al (2005) [38], [39], to be able to assess the additive and dominance effects separately, and to compare our numerical results to those obtained by e.g. Schmidt et al 2006 [21], who used the same notation. The data was restricted to the patients and non-AMD controls, since no smoking data was available from the blood donor controls. A series of models, which included the additive and dominance effects for each locus (*CFH* Y402, *LOC387715 A69S* and *C3* R102G) and environmental effects (sex, smoking) and various G×G, G×E and E×E interactions were fitted to the data. The non-nested models were compared by the means of Akaike's information criterion (AIC), where there is a difference of 2 or more between the AICs of the models including and excluding the term in question, respectively, is taken as evidence of a significantly better fit to the data (as suggested by North et al. 2005) [39]. The existence of dominance effects of individual loci could be ruled out. Thus, at the next stage, when the possible interactions were examined in more detail, only the additive genetic terms (and no dominance terms) were allowed for. This was also necessary in order to keep the number of parameters in the model to a reasonable number.

In addition to the logistic regression approach, which is known to have only modest powers for distinguishing interactions [40], [41], the possible interactions were further sought using two complementary approaches. i) Departure from the additivity model as described by Rothman [42] and implemented by Andersson et al. 2005 (www.epinet.se) [43]. Rothman has shown that independent risk factors adhere to an additive model where interaction is assessed based on departure from additivity of the disease rates. In this model, biological interaction (that is different from the term biological interaction in cell biology) is assessed using three measures: RERI (the relative excess risk due to interaction), AP (the attributable proportion due to interaction) and S (the synergy index) [43]. A synergy index exceeding 1.00 suggests the presence of at least one shared (metabolic) pathway in the pathogenesis of the disease, where both of the risk factors are required. ii) the mutual information-based statistics for testing interaction between two unlinked loci. Mutual information statistics is designed to measure the dependence between two random variables that can be detected by testing their independence (Methods S1) [44]. Logistic regression analyses and synergy index were calculated either with the SPSS (SPSS Inc., Chicago, Illinois; release 15.0, 2006) statistical software or with R language. We considered two loci G_1_ and G_2_, with each locus having two alleles.

## Results

### Individual genetic effects

The AMD-associated *LOC387715* A69S risk allele *T* was over-represented in our patient material with the *T* allele frequency reaching 48% in AMD cases compared to 19.5% in non-AMD controls (*p* = 3.75×10^−9^) and 24.7% in blood donor controls (*p* = 2.78×10^−19^) (Tables S2, S3 and S4). Both familial and sporadic cases also carried the LOC387715 risk genotype *TT* more often than the *GG* genotype (compared to the non-AMD controls: *p* = 2.73×10^−12^ ; familial and *p* = 9.95×10^−7^; sporadic cases or compared to the blood donor controls : *p* = 1.35×10^−15^ ; familial and *p* = 8.48×10^−7^; sporadic cases). The difference between the genotype frequencies in the familial and sporadic cases (Table S2) was not statistically significant (*p* = 0.09).

We also analyzed the *HTRA1* rs 11200638 polymorphism (Table S2). *LOC387715* rs10490924 and *HTRA1* rs11200638 are located only 6.1 kb from each other, and correspondingly, we observed only 5 genotypes out of 787 that were different among these two variants. This is inconsistent with perfect LD between the two SNPs. As there is accumulating evidence for LOC387715 A69S to be the actual causal variant in AMD [32], [33], we focused on the association analyses of the LOC387715 gene in this study.

The *C3* R102G variant corresponding to the electrophoretic protein variant C3F (fast), was associated with AMD in familial cases when compared to non-AMD controls (*p* = 0.008) or to blood donor controls (*p* = 0.039) (Tables S2, S3 and S4). The heterozygous risk genotype *CG* was also detected more often in familial cases than in non-AMD or blood donor controls with *p*-values of 0.013 and 0.049, respectively. However, the difference in the frequency of the homozygous risk genotype *GG* between cases and controls did not to reach statistical significance since there were too few *GG* cases in our material. However the trend is clear: there seems to be a higher OR for homozygotes than for heterozygous cases.

The estimated population attributable risks (PAR) for a carrier of the risk allele of *CFH*Y402H, *LOC387715* A69S and *C3* R102G (using blood donor controls as a reference group) were 58.2%, 51.4%, and 5.8%, respectively. The joint population attributable risk for the three loci was 65.4%. Since carrying a risk allele in one locus does not exclude also carrying a risk allele in the other locus the summary PAR is less than the sum of the three single PARs. The PAR for smoking was 47.7%, note, however that it had to be estimated using non-AMD controls as a reference group; thus it cannot be interpreted as a true population-wise figure. Also, it is not directly comparable to the gene-wise PAR:s given above due to different reference group used.

### Smoking

Odds ratio for smoking was 3.22 (95% CI 1.81–6.09, *p* = 4.69×10^−5^, non-AMD controls), and the joint OR for the three loci and smoking was 74.3 (95 %CI 10.81–2123.6, *p* = 1.54×10^−7^, non-AMD controls).

### Joint OR:s

We also assessed the joint OR:s of *complement factor H* Y402H (*CFH*) polymorphism, previously shown to be associated with AMD in the Finnish population [16], and *LOC387715* A69S polymorphism. Joint analysis of ORs for the two variants showed that the risk of AMD was 27-fold (*p* = 1.66×10^−12^, blood donor controls) if an individual had both homozygous risk genotypes, *CC* (*CFH*) and *TT* (*LOC387715*), compared to the non-risk genotype *TTGG,* respectively, with all the other joint OR:s ranging from 2 to 21 (Table 1). Three-locus risk-allele carrier (*CFH* Y402H, *LOC387715* A69S and *C3* R102G) joint OR:s are given in Table 2. The risk of AMD was 18-fold for a carrier of at least one risk allele per susceptibility loci when compared to a non-carrier of risk alleles at any loci in blood donor controls (Table 2).

**Table 2:** Three-locus risks (odds ratios [OR], 95% confidence intervals [95%CI], and *p*-values) for combinations of the risk allele carriers and non-carriers of the *complement factor H* (*CFH*) Y402H, the *LOC387715* A69S and the *complement component 3* (*C3*) R102G polymorphisms.

At least one risk allele present in:	OR	(95%CI)	*p*-value
None	1.00		
*CFH* (C)	5.45	(2.18–16.83)	1.26×10^−4^
*LOC387715* (*T*)	4.89	(1.73–16.43)	3.72×10^−3^
*CFH* (C) and *LOC387715* (*T*)	15.55	(6.42–47.22)	1.42×10^−12^
*C3* (*G*)	2.12	(0.52–8.70)	0.296
*CFH* (C) and *C3* (*G*)	5.57	(2.14–17.72)	2.75×10^−4^
*LOC387715* (*T*) and *C3* (*G*)	12.59	(3.63–50.89)	3.62×10^−5^
*CFH* (C) and *LOC387715* (*T*) and *C3* (*G*)	17.91	(7.12–55.88)	1.70×10^−12^

Seven combinations are compared to individuals who carry no risk allele. Risk alleles are *C* (*CFH*), *T* (*LOC387715*) and *G* (*C3*). Patients with AMD (n = 332) are compared to blood donor controls (n = 350).

### Interaction analyses

In the logistic regression modelling, we found highly significant (*p*<0.001) additive gene effects for the *LOC387715* and *CFH* loci, both having approximately the same effect size (Table 3). Also, for *C3* an additive effect was seen (*p* = 0.007), which was slightly weaker than the individual effects of *CFH* and *LOC387715,* but still made the overall fit of the three-locus model statistically significantly better than any two-locus models. No dominance effect could be demonstrated for either *CFH* Y402H, *LOC387715* A69S, or *C3* R102G.

**Table 3:** Final model parameters from the logistic regression analyses: the logarithm of odds of being a case rather than a control is explained by genetic effects and their interactions and a set of covariates (for details, see text), from which an automatic stepwise procedure selects the final set of statistically significant variables and estimates their magnitudes.

Variable	*p*-value	Exp(b)	95% CI for Exp(b)
*CFH* additive effect	0.000	5.774	3.225–10.339
*LOC387715* additive effect	0.000	5.537	3.250–9.434
*C3* additive effect	0.003	2.421	1.346–4.355
*CFH* additive×*LOC387715* additive	0.057	1.945	0.980–3.863
Sex×smoking	0.065	1.684	0.967–2.931
*C3* additive×sex×smoking	0.016	0.429	0.216–0.852
Constant	0.000	6.078	

Exp(b) is the coefficient corresponding to a one unit change in the explanatory variable. Nagelkerke R Square for the final model was 0.431 (n = 382).

**Figure 1:**
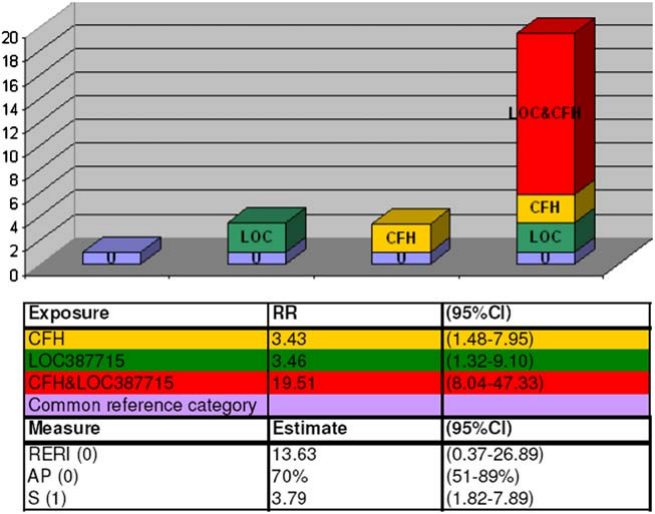
Measures of biological interactions between *LOC387715* A69S (LOC) and *CFH* Y402H (CFH). Regression coefficients for the calculations of the estimates were obtained from a separate logistic regression model as described by Andersson et al., 2005 [43] www.epinet.se. Figures in parenthesis after measures indicates values with no interaction.

Interestingly, an interaction between *CFH* and *LOC387715* was suggested (*p* = 0.057), with the estimated effect being only a bit smaller than the additive effect of *C3* locus alone. With the departure-from-additivity model the attributable proportion due to interaction of the loci was 70% (95%CI 51–89%) and S, the synergy index 3.79 (95%CI 1.82–7.89) (Fig 1). Also, mutual information-based (MI) statistics [44] resulted in a p-value of 1.69×10^−6^ supporting the results obtained with logistic regression analysis and the departure-from-additivity model (Table S5). No evidence for G×E interaction was obtained with another major susceptibility gene *LOC387715* and smoking (p = 0.14) whereas the G×E interaction with sex (p = 1.04×10^−6^) was shown using MI statistics. Here the higher number of female patients in our study material might affect the result.

No evidence for an independent effect of smoking could be detected in the logistic regression modelling in our data (*p* = 0.795). Instead, E×E and G×E interactions were suggested: sex×smoking (*p* = 0.043) and *C3*×smoking (*p* = 0.086). When combined into a 3-way interaction *C3*×sex×smoking (*p* = 0.016) the 2-way interaction *C3*×smoking disappeared. The sex×smoking interaction term preserved borderline significance (now, *p* = 0.065) (Table 3). Based on the difference in the AICs of the model including the two 2-way terms vs. one 3-way interaction term proved the latter to be more parsimonious (data not shown). However, synergy index and mutual information statistics (Table S5) failed to show evidence for interaction between neither sex, smoking nor *C3.* Possible explanations for this complexity in the logistic regression modelling showed up in further stratified analyses, where we found out that i) ever-smoker women had 4.68-fold (95%CI 1.95–14.12, *p* = 3.60×10^−4^) risk of AMD when compared to never-smoker women, whereas in men the odd's ratio was smaller (OR = 2.57) and of borderline significance (95%CI 0.99–6.86, *p* = 0.054). Thus, the effect of smoking was more pronounced in women, which also explains the significance of the sex×smoking interaction. At the same time it explains why no independent main effect for smoking alone was obtained. ii) *C3*×smoking: smoking seemed to have a significant risk-conferring effect on the non-risk genotype *CC*, but the effect is not that strong (and non-significant) in *G*-carrying genotypes (although the trend is the same). In the never-smokers, the *C3 G* allele predisposed to AMD as expected, whereas, interestingly, in ever-smokers the effect of *G* allele was virtually indistinguishable. iii) *C3*×sex×smoking: here we saw the highest OR for ever-smoker women with the non-risk genotype *CC* (OR = 8.55, [95%CI 2.45–58.46], *p* = 0.0002), and non-significant effects in *G*-carrying genotypes (though the trend is the same, that is, smoking predisposed to AMD in all genotype×sex classes, OR:s around 2). Overall, both smoking and carriership of the *G* allele confers risk for AMD, but the effect could be mediated via a different pathway.

## Discussion

Here we report joint risks of three gene (*CFH*, *LOC387715* and *C3)* loci, and smoking in AMD. We found tentative evidence for a gene-gene interaction between *CFH* and *LOC387715,* the two major susceptibility genes of AMD using three different statistical approaches. The first evidence for interaction was obtained using logistic regression; however, because the power of logistic regression to detect interactions is low [40], it *de facto* necessitated the use of other, more sensitive methods. Thus we applied the departure-from-additivity model that is based on additive disease rates [42], [43]. This method has also been used by Despriet et al [45] who reported increased risks of AMD in *CFH* risk genotype carriers with high C-reactive protein serum levels, elevated sedimentation rate (ESR), leucocyte count and smoking and more recently by Baird et al., [46] who demonstrated a G×E–interaction between a pathogenic load of C. pneumoniae and the *CFH* Y402H in the aetiology of AMD. The third method, mutual information statistics, has not been previously utilized in analyzing risk factors for AMD also suggested strong evidence for interaction of *CFH* and *LOC387715.*

The strong association of the *LOC387715* A69S with AMD in our Finnish patient material is in agreement with previous results from other populations [11], [15], [21]–[24], [32], [47]–[51] (Table 4). Furthermore, joint risks for *CFH* Y402H and *LOC387715* A69S were also of similar magnitude as observed by Rivera et al., [11]. In addition, the joint additive effect of *CFH* and *LOC387715* has been shown to be strengthened by rare C2 variants [51]. However, discoveries of interactions between these loci (or smoking) have been scarce and they have not been replicated. Thus, we want to point out that most of the analyses of G×G and G×E interaction have been based on logistic regression modelling [11], [15], [21], [22], [47], which according to our present state of understanding is statistically not a very powerful approach [40], [41]. Thus, re-analysis of existing data with more sensitive methods for detecting interaction might, possibly, change the picture a bit. Indeed, a true biological interaction between *CFH* and *LOC387715* would be plausible based on recent cell biological data by Kanda et al. (2007) and Fritsche et al. (2008) showing that *LOC387715* is localized to mitochondria [32], [33] and on the earlier evidence that mitochondria activate complement [52]. Interestingly, mitochondrial dysfunction involving altered mitochondrial translation, import of nuclear-encoded proteins, and ATP-synthase activity was suggested in recent proteomics studies on retinal pigment epithelium (RPE) in AMD by age [53]. Further cell and neurobiological studies are warranted to elucidate whether the *CFH* and the *LOC387715* genes indeed belong to a common causative pathway.

**Table 4:** Table summarizes odds ratios (OR) and 95% confidence intervals (CI) of our current study and previous studies on the *LOC387715* A69S polymorphism.

Study	AMD cases (n)		Controls (n)	OR_het_ (95% CI)	OR_hom_ (95%CI)
This study		332	105	3.37 (2.09–5.52) cases compared to 105 non-AMD controls	17.69 (6.23–76.92)
			350	2.77 (1.98–3.89) cases compared to 350 blood donor controls	7.39 (4.42–12.77)
Rivera et al. 2005		1120	922	2.69 (2.22–3.27)	8.21 (5.79–11.65)
Schmidt et al. 2006		610	259	1.65 (1.12–2.43)	5.73 (3.07–10.71)
Conley et al. 2006	AREDS sample	701	175	3.06 (2.13–4.39)	17.26 (6.22–47.89)
	CHS sample	126	1051	1.58 (1.05–2.39)	4.75 (2.56–8.80)
Tanimoto et al. 2007		95	99	2.15 (1.16–4.03)*	
Ross et al. 2007	NEI sample+ AREDS sample	399	329	2.61 (1.89–3.61)	8.59 (4.49–16.46)
	BMES sample	278	557	1.69 (1.25–2.28)	2.20 (1.05–4.62)
Kanda et al. 2007		535	288	2.66†	7.05†

OR_het_ refers to comparison of *GT* to *GG* and OR_hom_ to comparison of *TT* to *GG*. *T* allele is the risk allele.

AREDS Age-Related Eye Disease Study

CHS Cardiovascular Health Study

* OR for individuals having one or both risk alleles (T)

NEI National Eye Institute

BMES Blue Mountains Eye Study

† relative risk

A G×E interaction between *CFH* Y402H and smoking could be detected in our material using MI statistics (p = 8.24×10^−4^; Table S5). In the Rotterdam study increased risks of AMD in *CFH* risk genotype carriers with smoking, elevated C-reactive protein serum levels, sedimentation rate (ESR) and leucocyte count were reported using the departure from additivity model (synergy index of 2.38 [95%CI 1.06–5.35]) [45] concordant with our results. An interaction between smoking and *CFH* would be plausible, since cigarette smoke has been shown to activate the complement system [54]. Smoking also increases serum CRP concentrations [55] and further, impaired binding of C-reactive protein to the *CC* or *CT* variants of *CFH* (Y402H) has been detected [56], [57]. However, in studies using logistic regression analyses smoking and *CFH* Y402H have consistently been found to be statistically independent risk factors for AMD [15], [58]–[60].

We did not find an interaction between LOC387715 and smoking with any of the three statistical methods used that is in agreement with several previous findings [15], [23], [48], [60], although Schmidt et al. have reported a statistical interaction between LOC387715 and smoking [21] and most recently, an interaction between HTRA1 rs11200638 and smoking was reported [34].

Here we also demonstrate that *C3* seems to have a milder, though a statistically significant effect on AMD, as noted earlier [35], [36] (Table 5). Accordingly, the risk of AMD was 16-fold for a carrier of at least one risk allele at both *CFH* Y402H and *LOC387715* A69S loci, while adding the risk allele of the *C3* R102G increased the OR to only 18 using logistic regression analysis (Table 2). *C3* plays a crucial role in all the three pathways of the complement system, the classic, the lectin and the alternative pathway [37]. *C3* has also been identified in drusen deposits, the hallmarks of AMD [7]. Considering this, the G×G interaction of *C3* and CFH Y402H in AMD obtained using MI statistics is reasonable. Furthermore, activation of an alternative pathway of the complement system by cigarette smoke has been shown to be mediated by *C3* [61], [62]. Considering this it would be reasonable to think that a true *C3*×smoking interaction might have exist.

**Table 5:** Table summarizes odds ratios (OR) and 95% confidence intervals (CI) of our current study and previous studies on the *complement component 3* (*C3*) R102G polymorphism.

Study	AMD cases (n)		Controls (n)	OR_het_ (95% CI)	OR_hom_ (95%CI)
This study		332	105	1.64 (1.00–2.75) cases compared to 105 non-AMD controls	1.92 (0.61–8.82)
			350	1.21 (0.87–1.67) cases compared to 350 blood donor controls	1.30 (0.62–2.79)
Yates et al. 2007	English group	603	350	1.6 (1.2–2.2)	2.4 (1.3–4.4)
	Scottish group	505	351	1.8 (1.2–2.6)	2.9 (1.4–5.9)
Maller et al. 2007		1238	934	1.61	3.26

OR_het_ refers to comparison of *CG* to *CC* and OR_hom_ to comparison of *GG* to *CC*. *C* allele is the risk allele.

The population attributable risks (PAR) for the *CFH* Y402H (58.2%) and LOC387715 A69S (51.4%) are in line with other reports where PARs for *CFH* Y402H range from 43 to 68 % [5], [13], [15], [20], [21] and for *LOC387715* A69S from 36 to 57% [15], [20], [21]. Instead, the PAR for *C3* R102G in Yates et al. was higher (22%) [35] than what we obtained (5.8%). It is possible that the *C3* G allele effect is diluted in our data by the stronger effect of *CC* genotype in ever-smoking women (*C3*×sex×smoking). Schmidt et al. [21] also reported a PAR for smoking which was 20% and thus lower than ours (47.7%), but here it should be noted that we could not estimate the true population-based PAR due to a lack of smoking information in our blood donor controls, and hence ours may be an overestimate. However, the ever-smokers' risk is mediated through the non-risk genotype *CC* of the *C3* gene whereas in the never-smokers the risk effect comes from the reported risk allele warrants further studies.

In conclusion, analysis of gene-gene and gene-environment interaction of risk factors in AMD benefits from combination of several statistical methods and the available biochemical data.

## Supporting Information

Methods S1 (1.23 MB DOC)

Table S1 (0.04 MB DOC)

Table S2 (0.08 MB DOC)

Table S3 (0.05 MB DOC)

Table S4 (0.05 MB DOC)

Table S5 (0.04 MB DOC)
