# Supplementary material for: Multifactor Effects and Evidence of Potential Interaction between Complement Factor H Y402H and LOC387715 A69S in Age-Related Macular Degeneration
Source: PLoS One. 2008 Dec 2;3(12):e3833. doi: 10.1371/journal.pone.0003833 (PMC2585793; doi:10.1371/journal.pone.0003833)
Supplement: Methods S1 — (1.23 MB DOC) [file pone.0003833.s001.doc]

**Methods S1**

**Mutual Information-based Statistics for Testing the Interaction between Two Unlinked Loci**

We use mutual information that is widely used in communication and complex system analysis to measure gene-gene interaction. Consider two loci G_1_ and G_2_, each locus with two alleles. Mutual information is designed to measure the dependence between two random variables. The mutual information between two loci in the general population is defined as


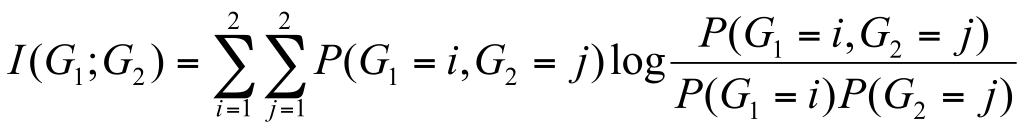
 (1)

Information theory (Cover et al. 1991) shows that mutual information
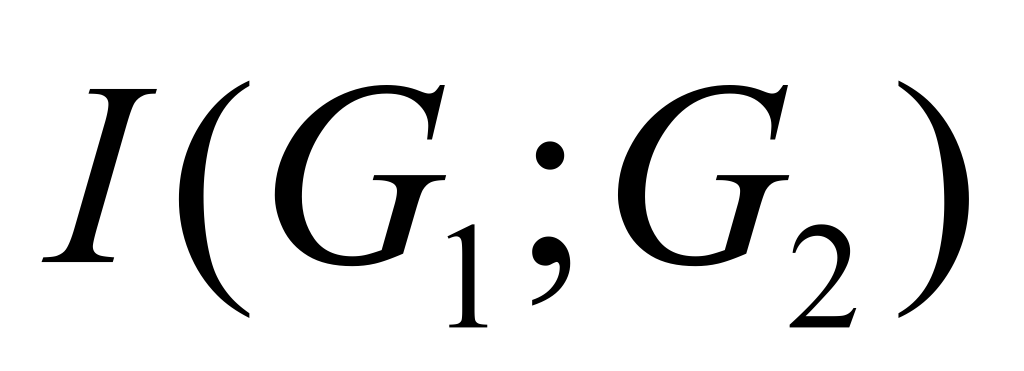
 is equal to zero if and only if


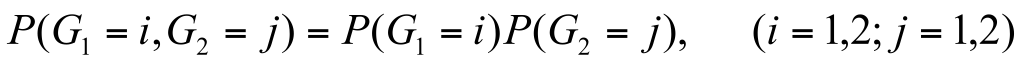


i.e., two random variables
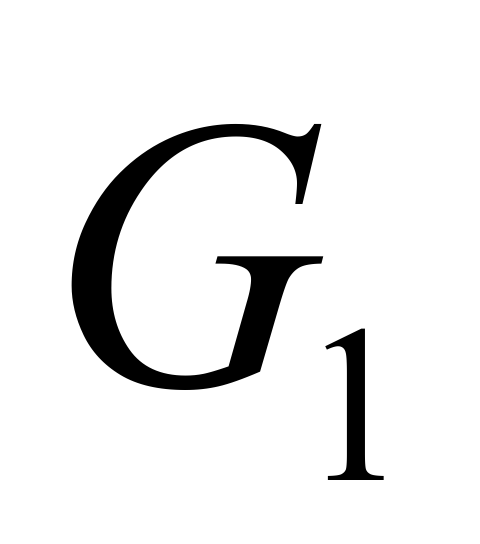
and
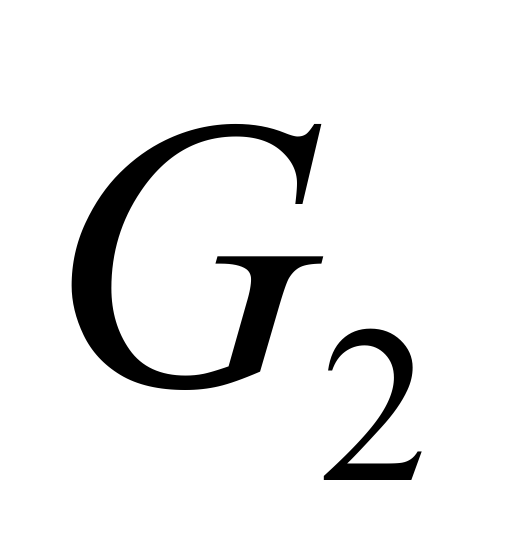
are independent.

We define the information measure of interaction between two unlinked loci as


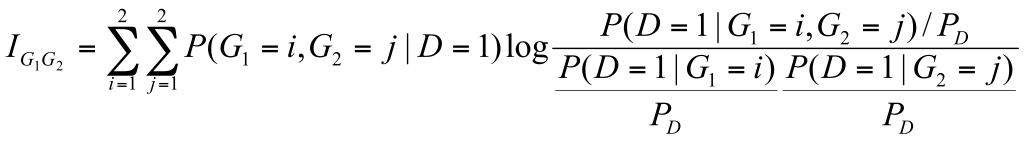
　　　　 (2)

which implies that
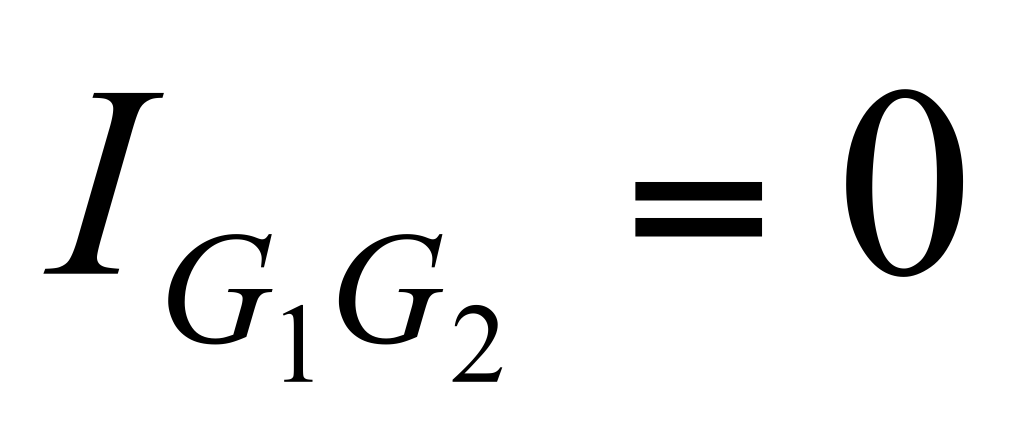
 if and only if


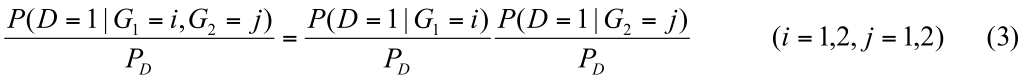


The information measure of interaction has two remarkable features. First, it is defined in terms of penetrance and hence related to the cause of the disease. Second, the interaction is measured by the interdependent operation of two loci in causing disease. The absence of an interaction between two loci indicates that equation (3) should hold.

If we assume that two variables
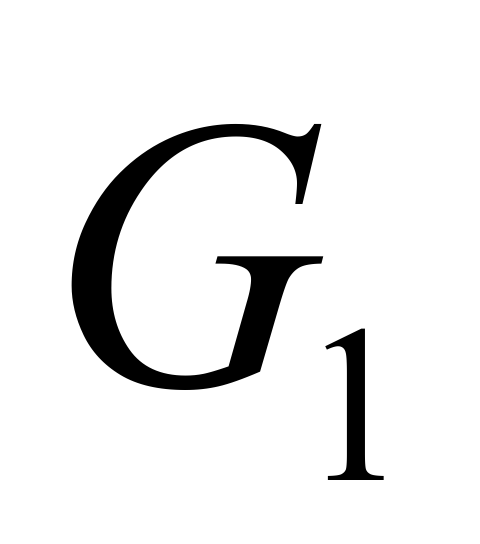
and
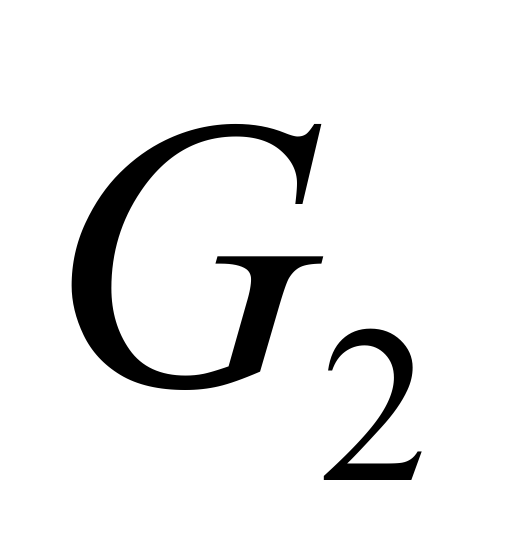
in the general population are independent, then


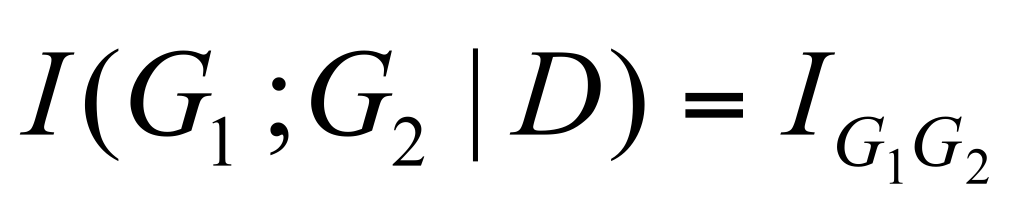


In this case, the mutual information between two loci in the disease population is equal to the information measure of the interaction between two loci. This provides an easy way to calculate the information measure of interaction between two unlinked loci.

Next we study the relationship between the information measure of interaction and the traditional odds ratio of interaction. Consider the following logistic model:


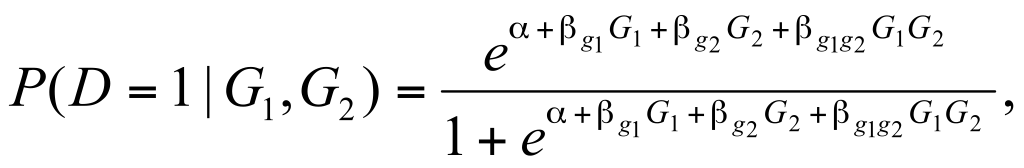


where
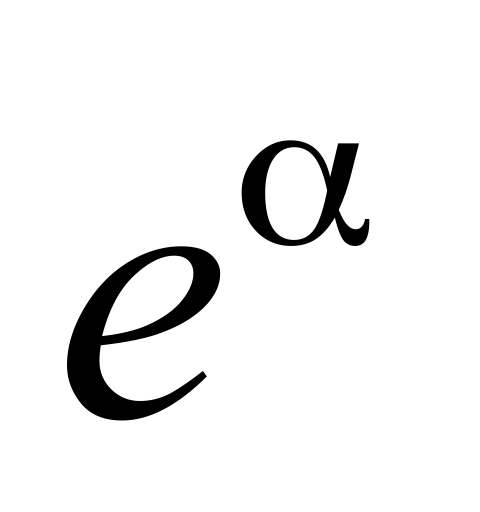
 is the baseline probability of the disease,
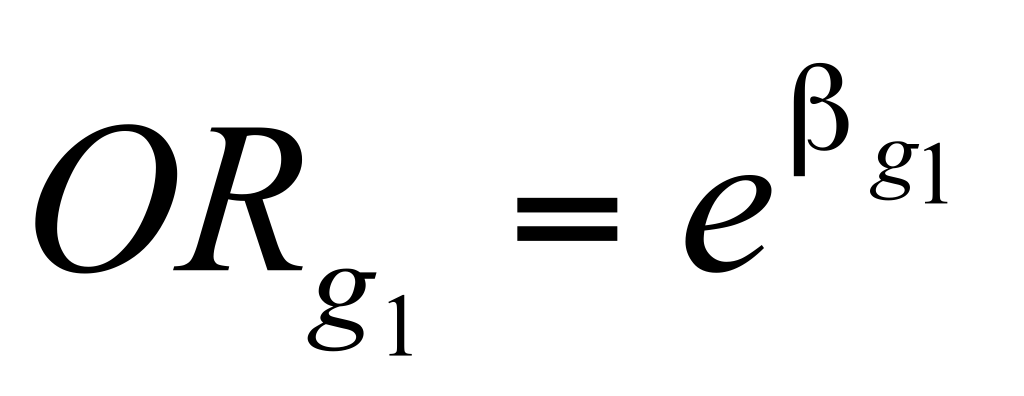
,
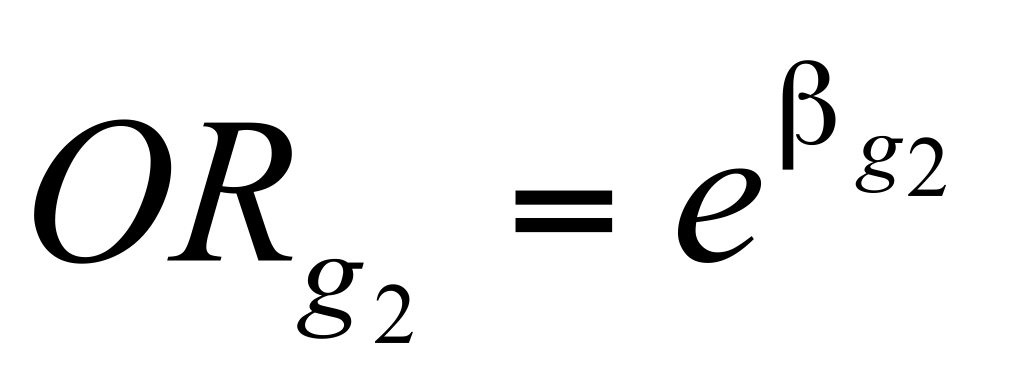
,
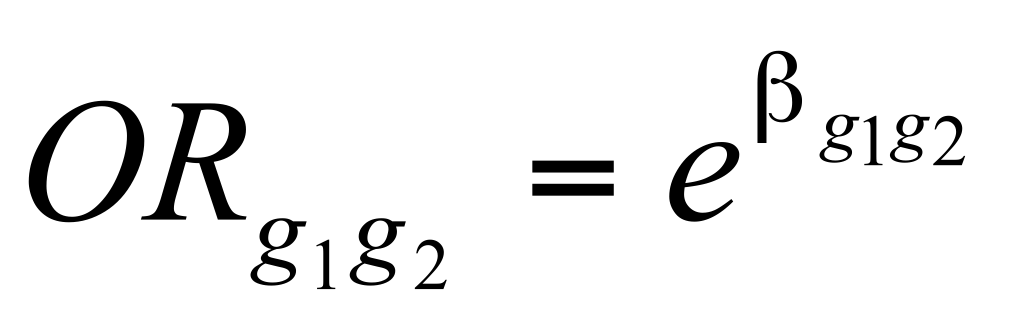
 are the genetic and interaction odds ratios, respectively.

We can show that if the information measure of interaction
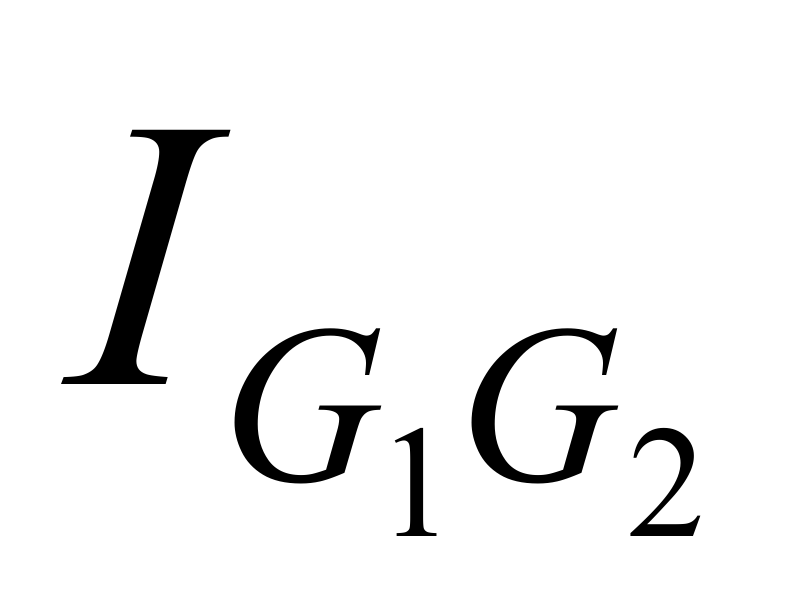
is equal to zero then we have


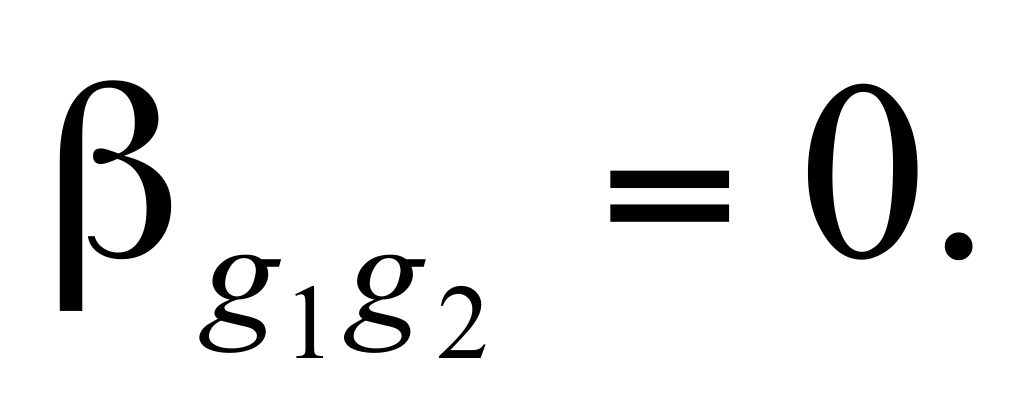


Interaction between two loci can generate the large difference in information measure of interaction between the general population and disease population, which motivated us to develop mutual information-based statistics for testing interaction between two unlinked loci.

The interaction between two unlinked loci can be detected by testing their independence or their differences in the components of the information measure of interaction between disease and general populations. Before defining the test statistic, we introduce some notations. Consider two loci G_1_ and G_2_, each locus with two alleles. Let


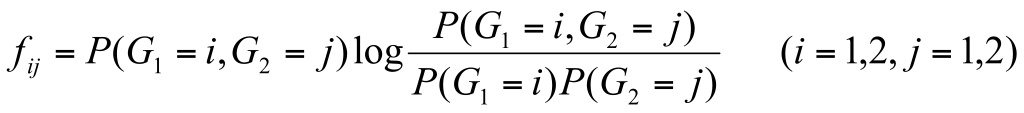


and
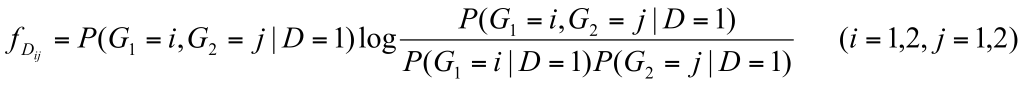
. Let
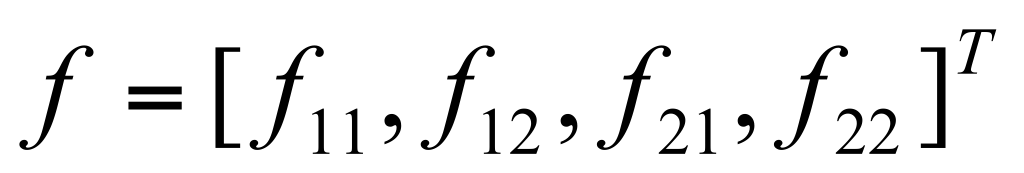
 and
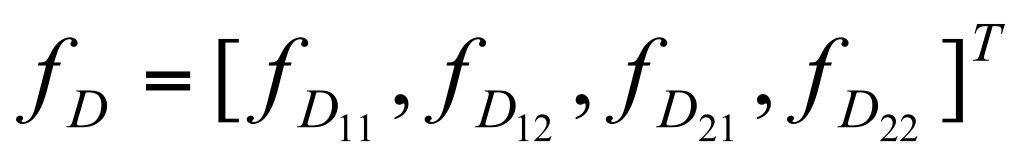


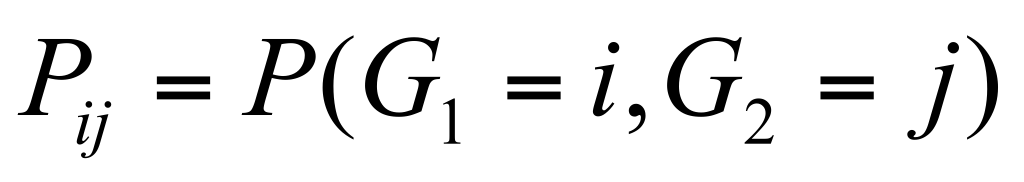
 and
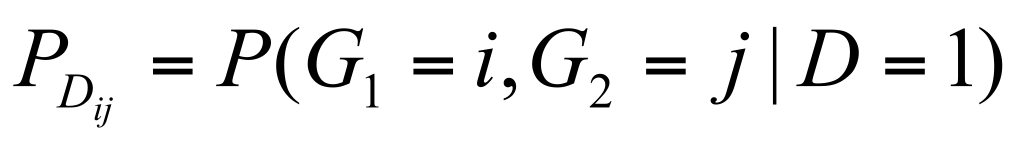
.

Define


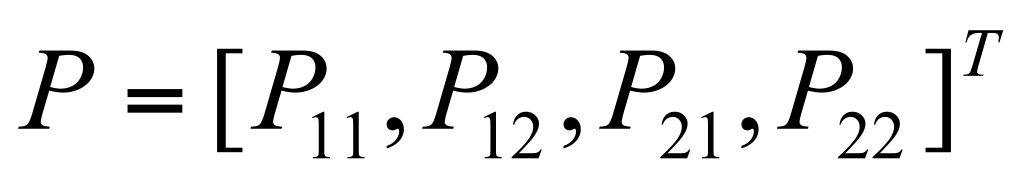
 and
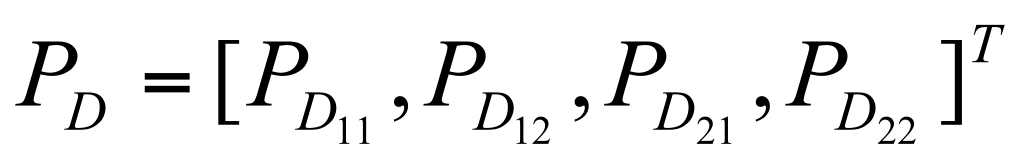
.

The joint probabilities of variables G_1_ and G_2_ in both the general population and the disease population follow multinomial distribution with the following covariance matrices.


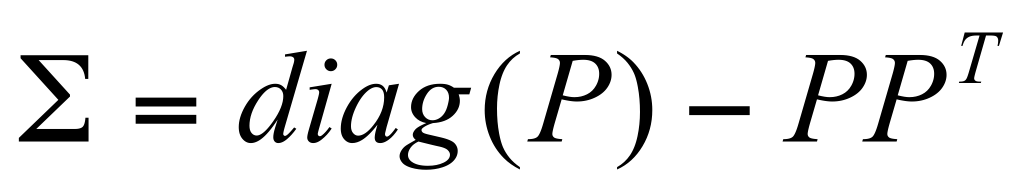
 and
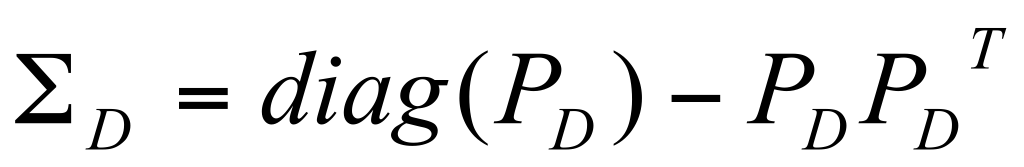
.

Let the Jacobean matrices of
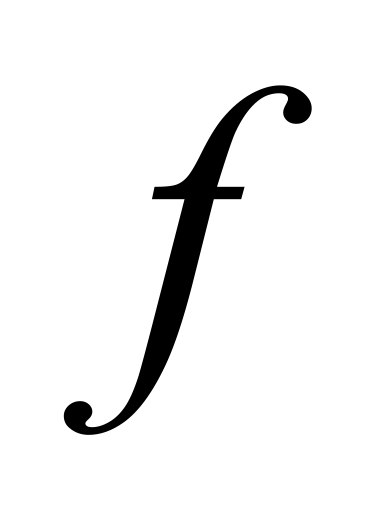
 and
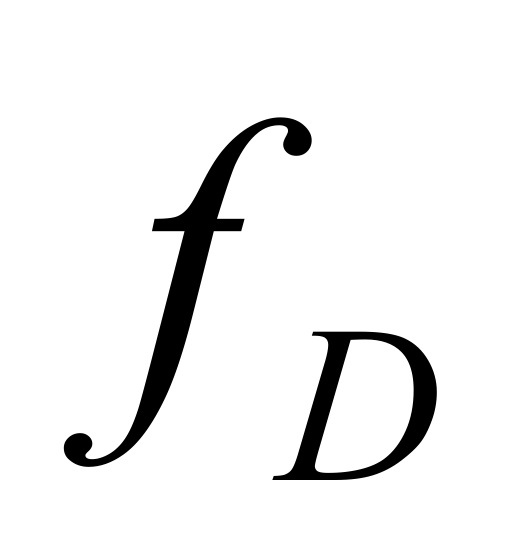
 with respect to
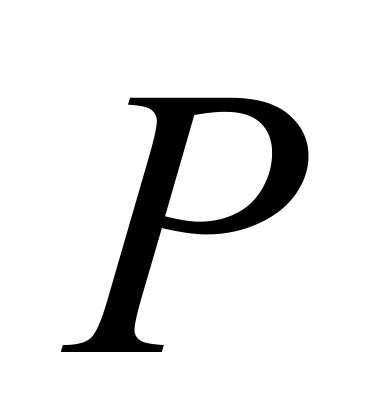
 and
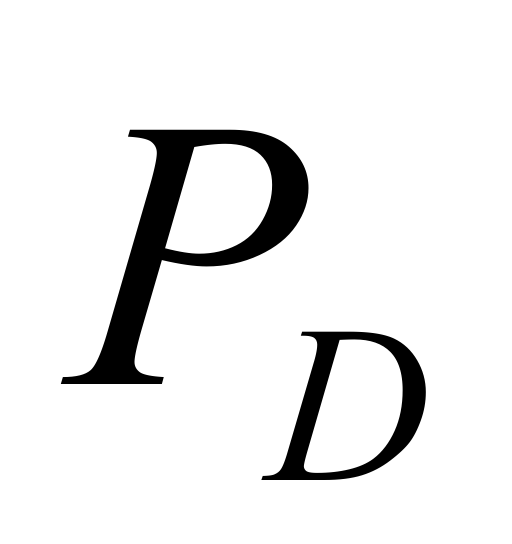
 be
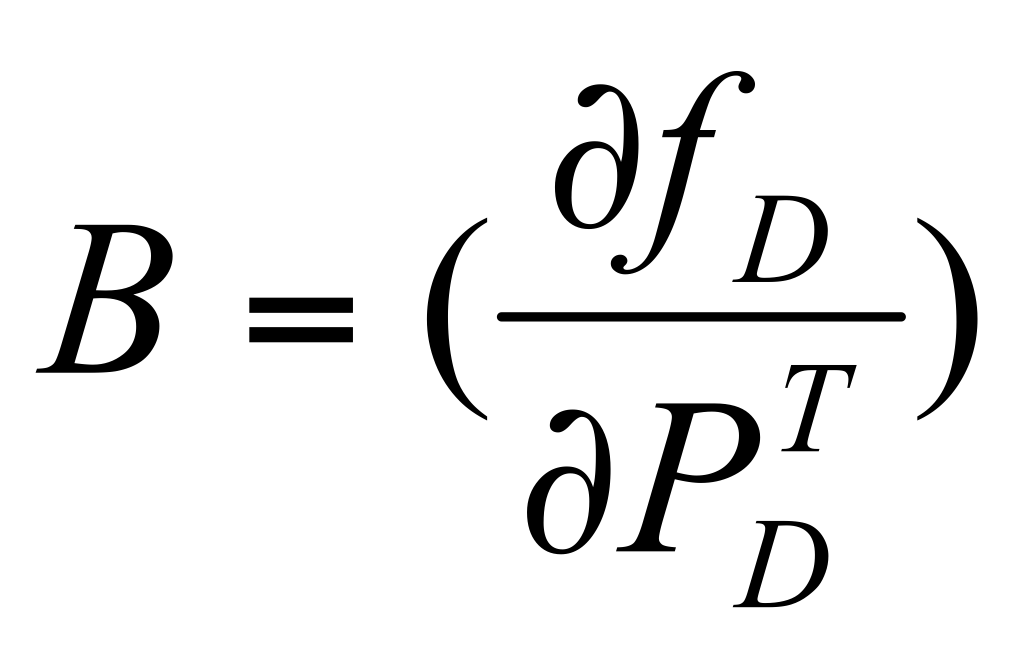
 and
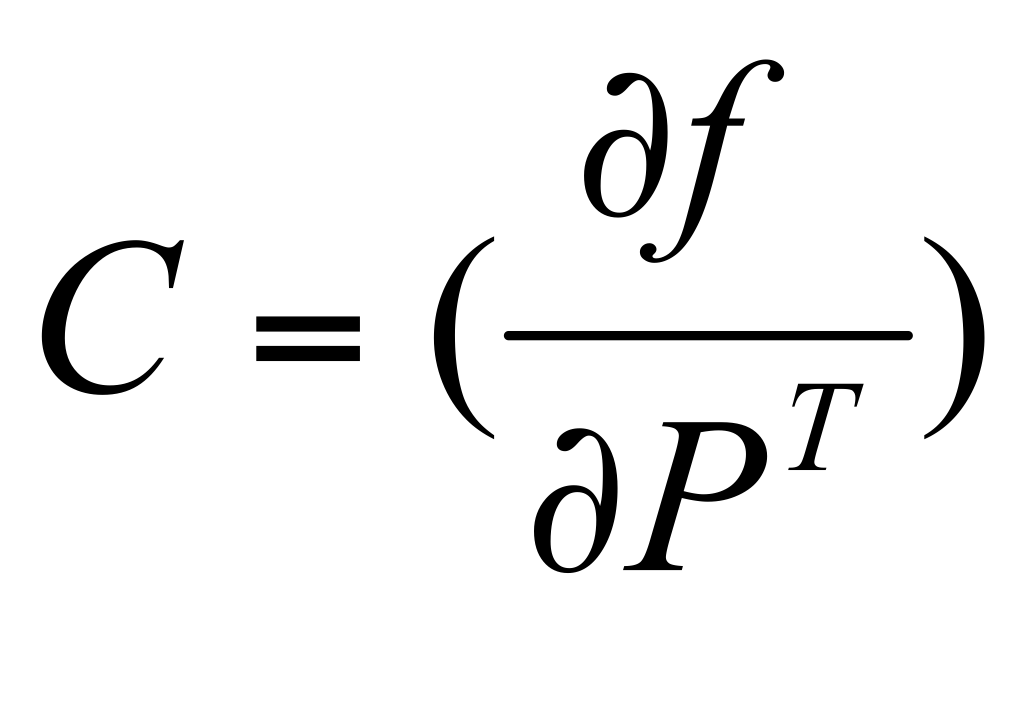
, respectively. It is easy to see that


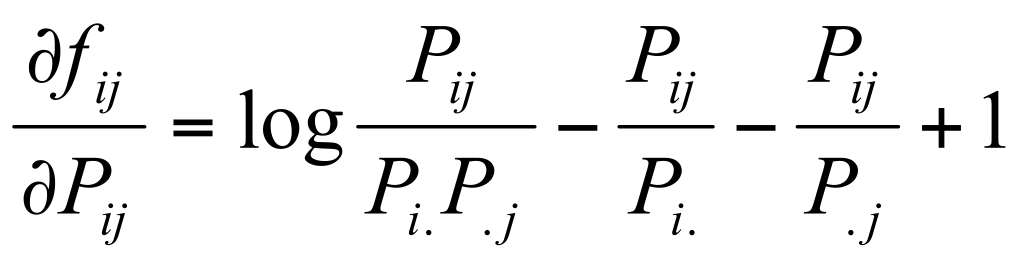
,
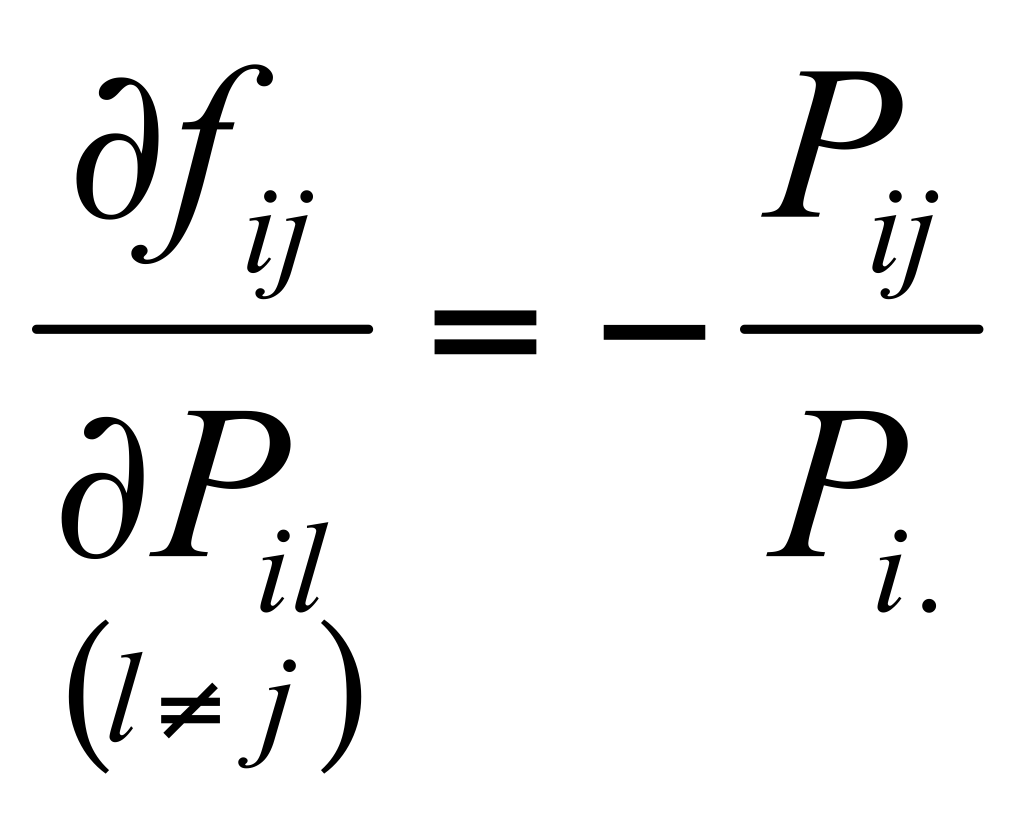
,
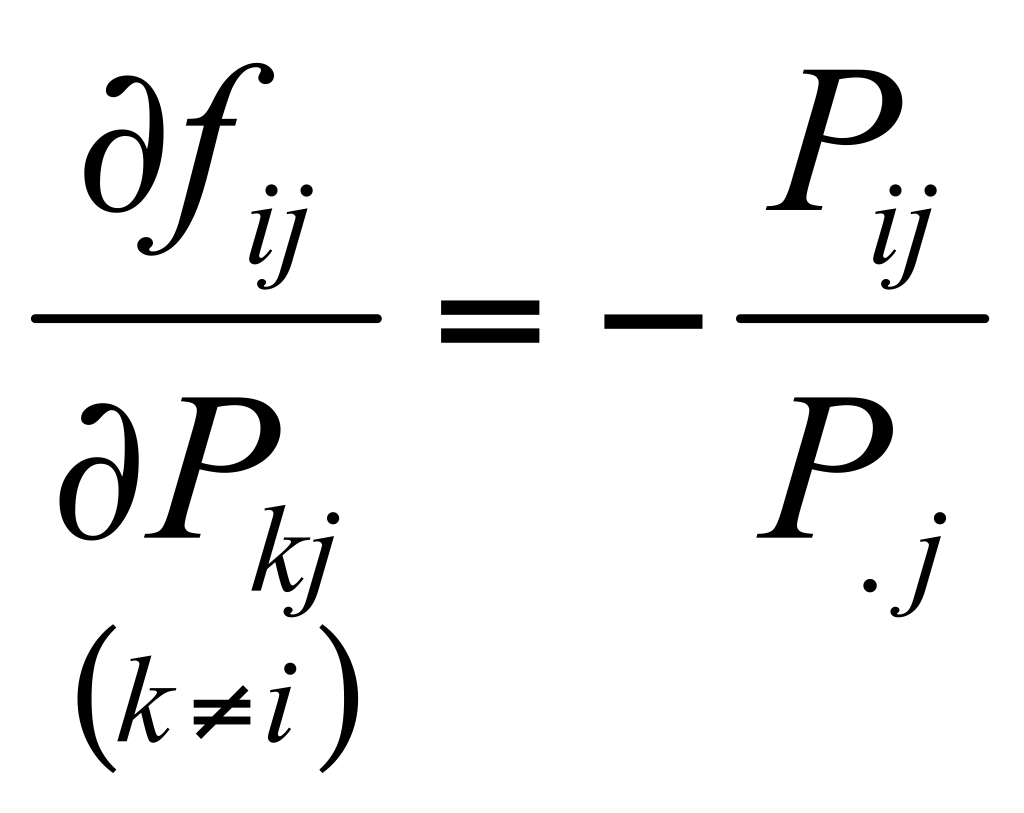
,
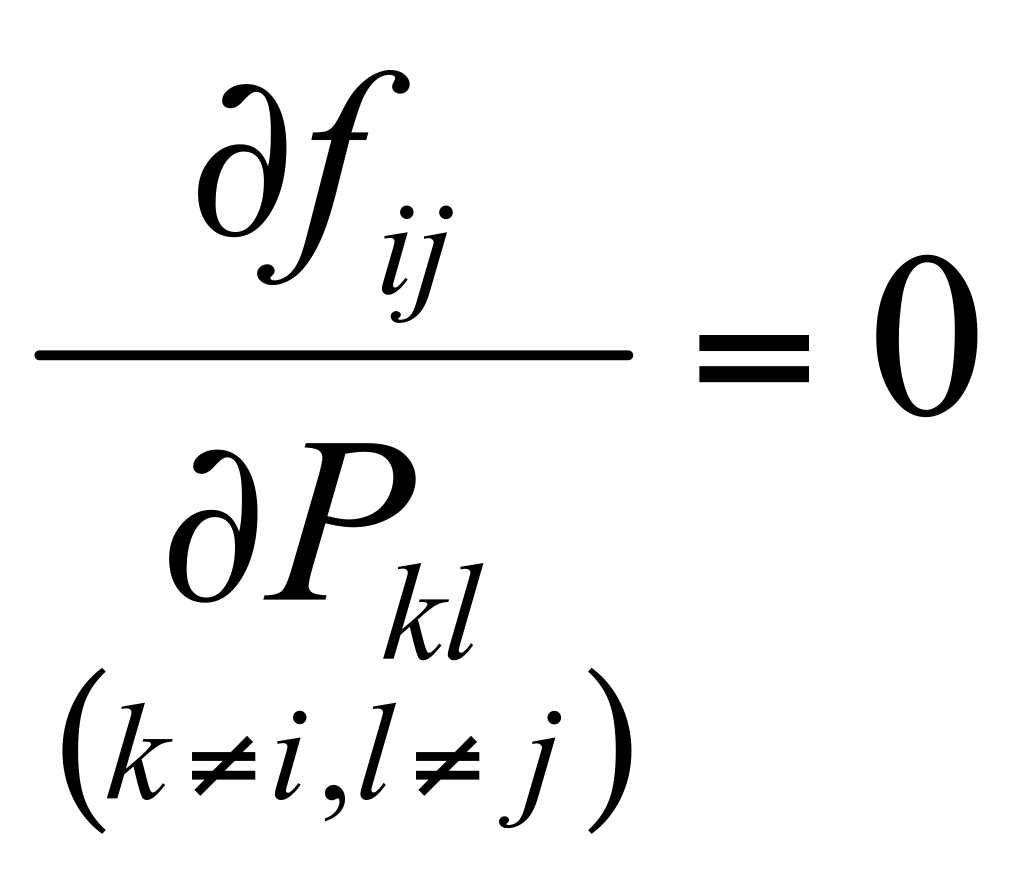


where
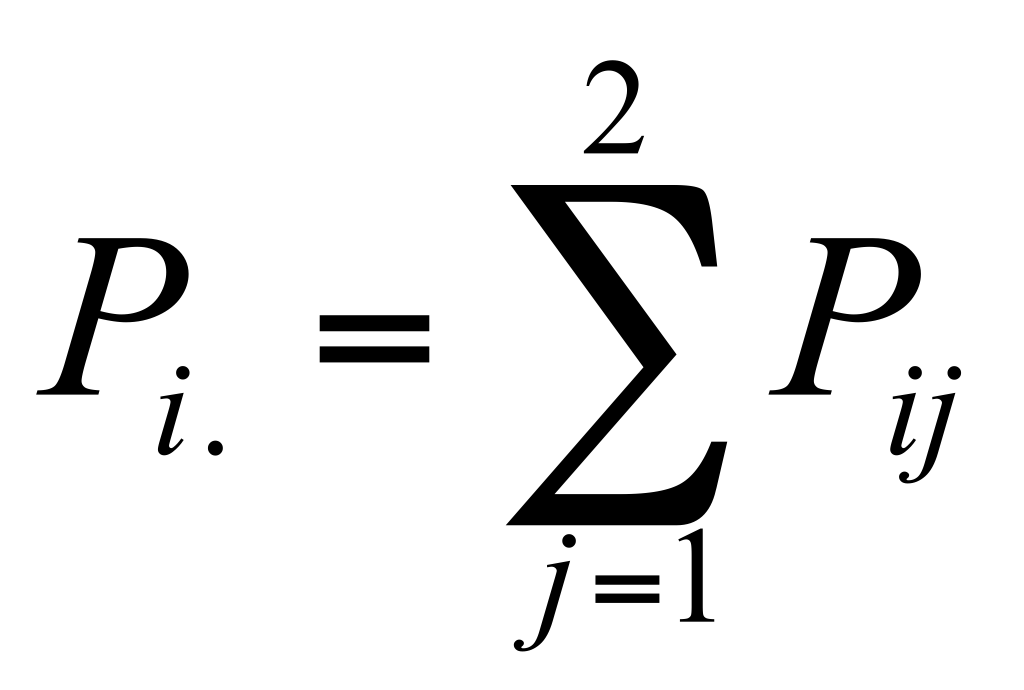
, and
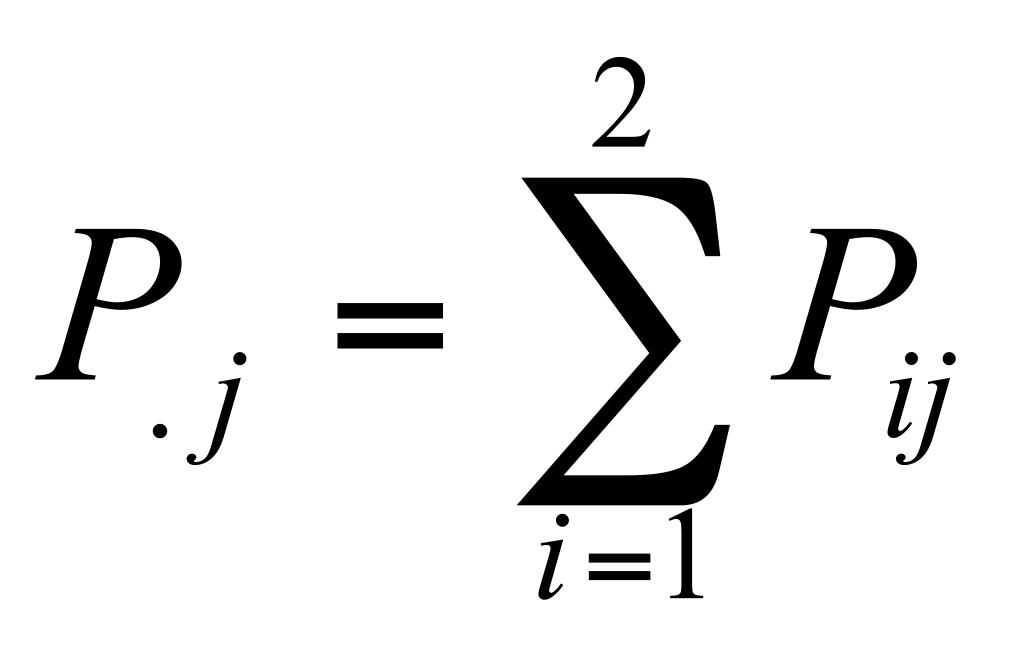
. The partial derivatives of the function
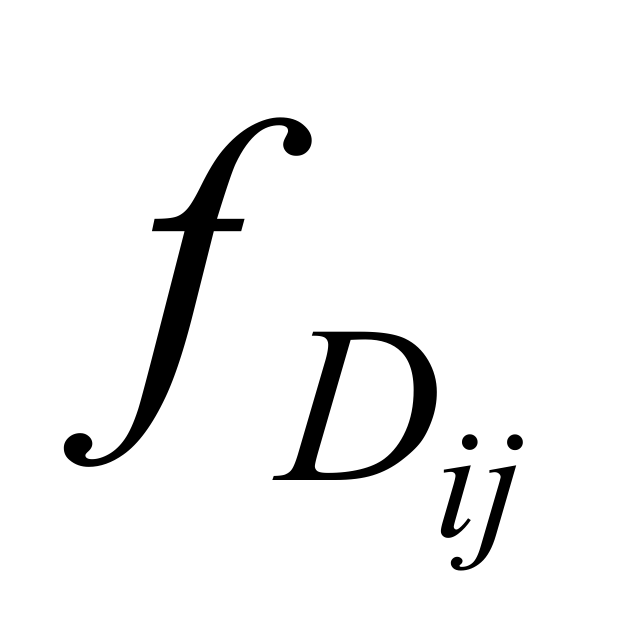
with respect to
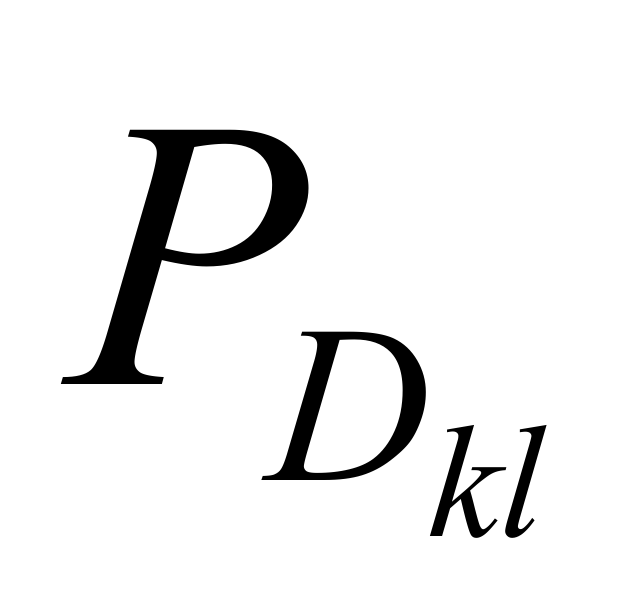
can be similarly defined. Let
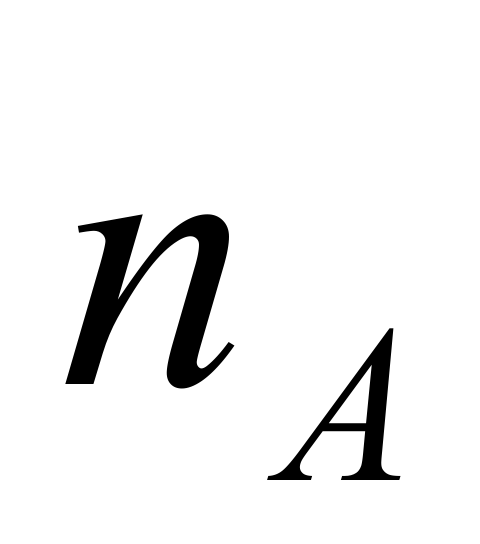
 be the number of sampled individuals in the cases and
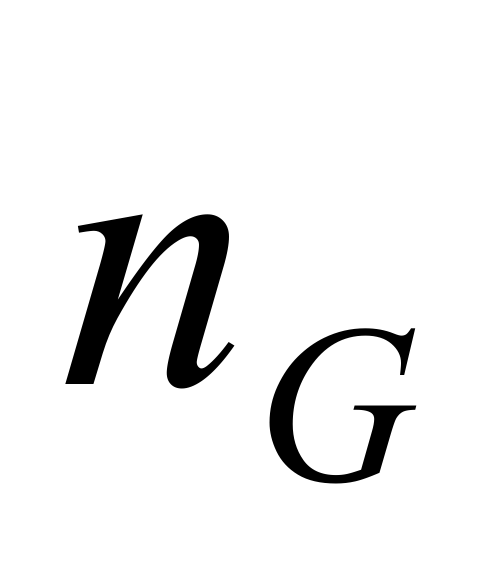
 be the number of sampled individuals in the controls. Define


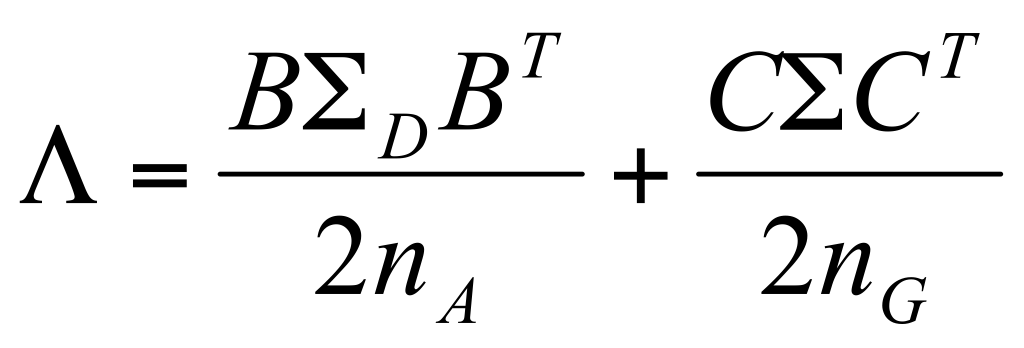
.

The statistic for testing interaction between two loci is then defined as


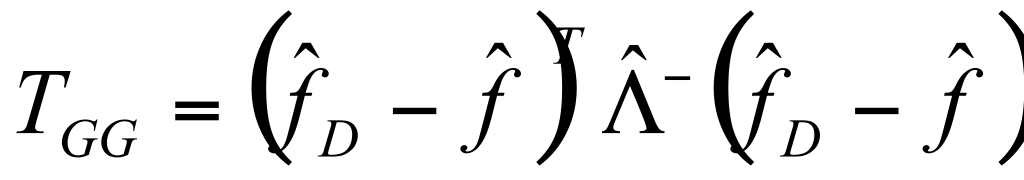


where
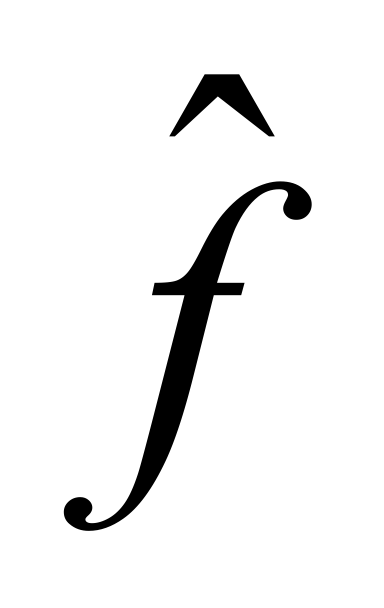
,
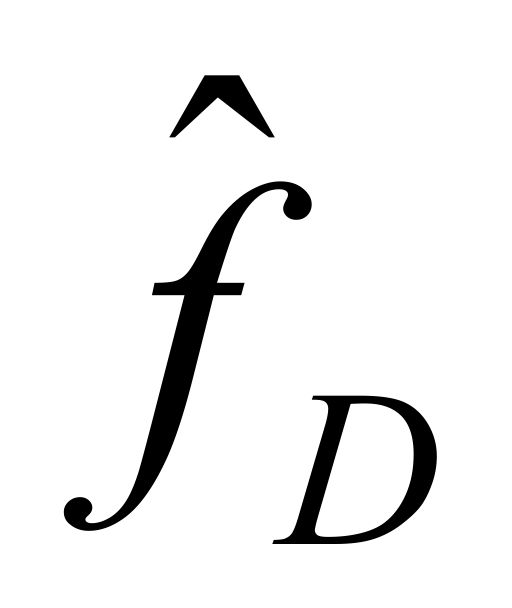
,
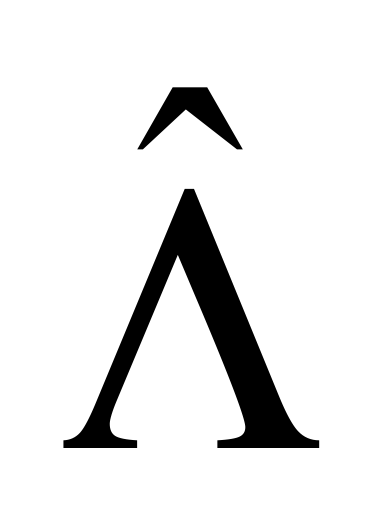
are the estimators of
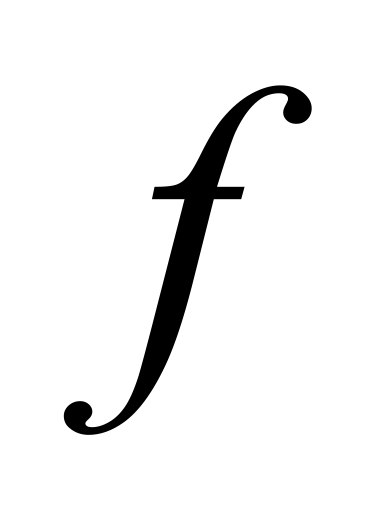
,
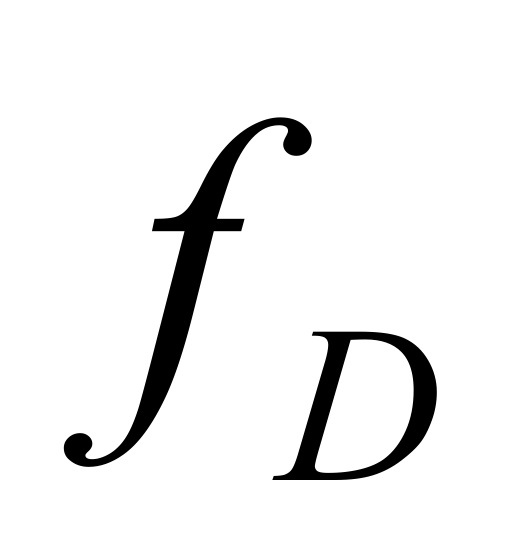
,
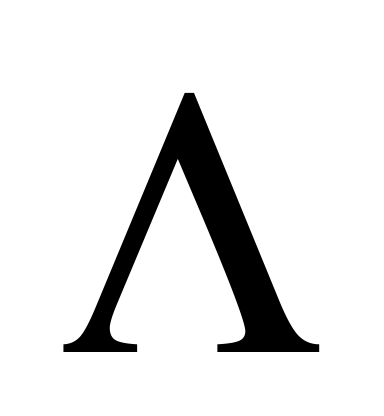
.
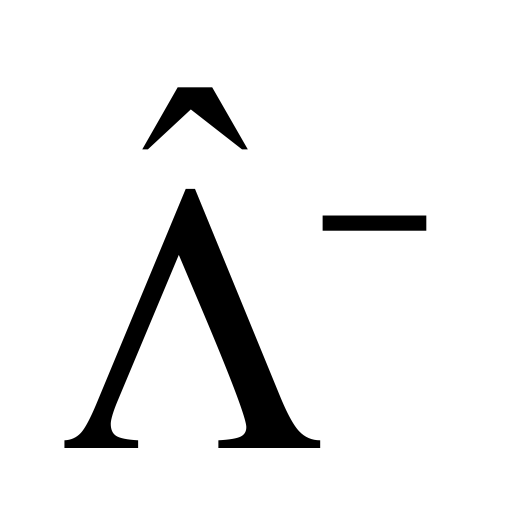
 is a generalized inverse of the

matrix
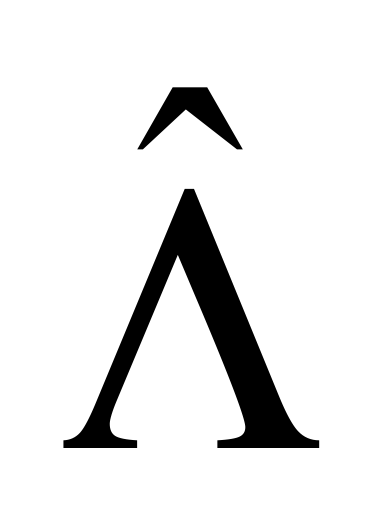
. When the sample size is large enough to ensure application of large sample

theory, the test statistic
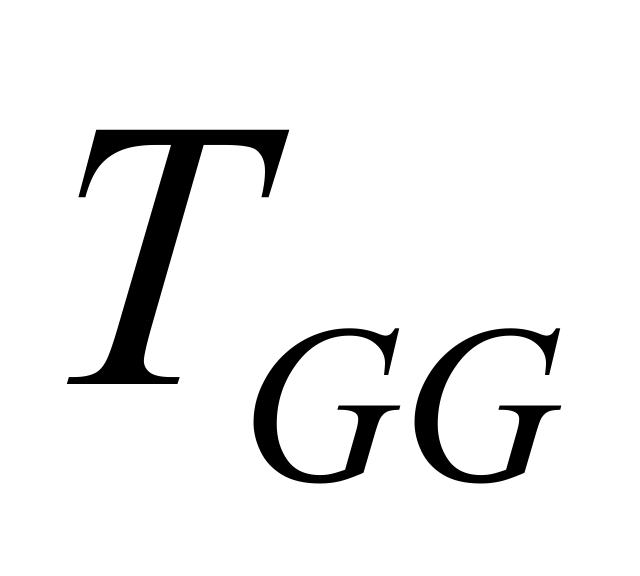
 is asymptotically distributed as a central
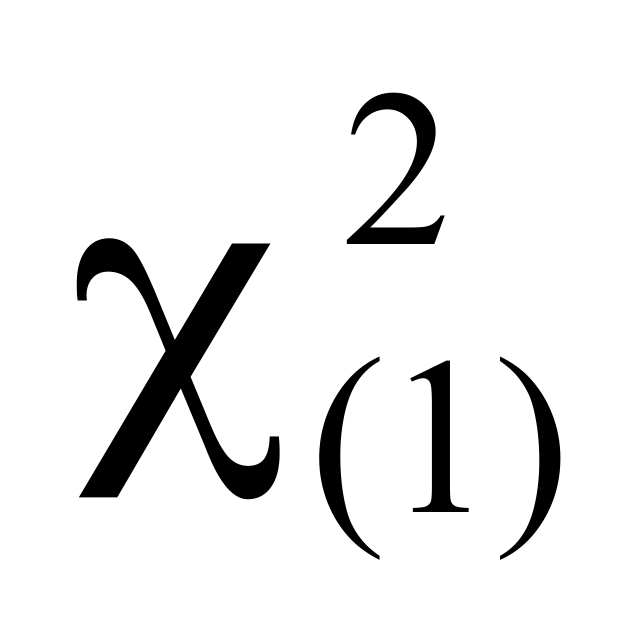
 distribution under the null hypothesis of no interaction between two loci, if we assume that variables G_1_ and G_2_ in the general population are independent.
