# Supplementary material for: Multifactor Effects and Evidence of Potential Interaction between Complement Factor H Y402H and LOC387715 A69S in Age-Related Macular Degeneration
Source: PLoS One. 2008 Dec 2;3(12):e3833. doi: 10.1371/journal.pone.0003833 (PMC2585793; doi:10.1371/journal.pone.0003833)
Supplement: Table S1 — (0.04 MB DOC) [file pone.0003833.s002.doc]

**Table S1. Clinical characteristics of the eyes of the patients in the study. For the type of the most advanced AMD lesion, the lesions were graded in the order of increasing severity: drusen, geographic atrophy involving the fovea and an acute exudative or a disciform lesion.**

| Patient group | N | Age mean | (range) | Large drusen | Geographic  atrophy  involving  fovea | Acute exudative or  a disciform lesion |
| --- | --- | --- | --- | --- | --- | --- |
| Sporadic AMD | 151 | 75.5 | (52.4-88.5) | 12.6% | 0.0% | 87.4% |
| Familial AMD  index patients | 106 | 75.8 | (56.3-92.7) | 17.0% | 12.3% | 70.7% |
| Familial AMD  relatives | 75 | 77.7 | (52.9-91.5) | 17.3% | 13.3% | 69.4% |
| Non-AMD  controls | 105 | 76.9 | (66.8-87.7) | 0.0% | 0.0% | 0.0% |
