# Supplementary material for: Multifactor Effects and Evidence of Potential Interaction between Complement Factor H Y402H and LOC387715 A69S in Age-Related Macular Degeneration
Source: PLoS One. 2008 Dec 2;3(12):e3833. doi: 10.1371/journal.pone.0003833 (PMC2585793; doi:10.1371/journal.pone.0003833)
Supplement: Table S2 — (0.08 MB DOC) [file pone.0003833.s003.doc]

**Table S2.** Allele and genotype frequencies (%) of A) the A69S polymorphism of the *LOC387715* gene (*T* allele is the risk allele), B) the promoter polymorphism of the *HTRA1* gene (*A* allele is the risk allele), and C) the R102G polymorphism of the *complement component 3* (*C3)* gene (*G* allele is the risk allele).

A)

| *LOC387715* | Familial cases (n=181) | Sporadic cases (n=151) | All AMD cases (n=332) | Non-AMD controls (n=105) | Blood donor controls (n=359) |
| --- | --- | --- | --- | --- | --- |
| Genotype |  |  |  |  |  |
| *TT* | 0.276 (n=50) | 0.179 (n=27) | 0.232 (n=77) | 0.029 (n=3) | 0.064 (n=23) |
| *GT* | 0.475 (n=86) | 0.510 (n=77) | 0.491 (n=163) | 0.333 (n=35) | 0.365 (n=131) |
| *GG* | 0.249 (n=45) | 0.311 (n=47) | 0.277 (n=92) | 0.638 (n=67) | 0.571 (n=205) |
|  |  |  |  |  |  |
| Allele |  |  |  |  |  |
| *T* | 0.514 | 0.434 | 0.477 | 0.195 | 0.247 |
| *G* | 0.486 | 0.566 | 0.523 | 0.805 | 0.753 |

B)

| *HtrA1* | Familial cases (n=181) | Sporadic cases (n=151) | All AMD cases (n=332) | Non-AMD controls (n=105) | Blood donor controls (n=350) |
| --- | --- | --- | --- | --- | --- |
| Genotype |  |  |  |  |  |
| *AA* | 0.282 (n=51) | 0.185 (n=28) | 0.238 (n=79) | 0.029 (n=3) | 0.063 (n=22) |
| *GA* | 0.470 (n=85) | 0.503 (n=76) | 0.485 (n=161) | 0.333 (n=35) | 0.377 (n=132) |
| *GG* | 0.249 (n=45) | 0.311 (n=47) | 0.277 (n=92) | 0.638 (n=67) | 0.560 (n=196) |
|  |  |  |  |  |  |
| Allele |  |  |  |  |  |
| *A* | 0.517 | 0.437 | 0.480 | 0.195 | 0.251 |
| *G* | 0.483 | 0.563 | 0.520 | 0.805 | 0.749 |

C)

| *C3* | Familial cases (n=181) | Sporadic cases (n=151) | All AMD cases (n=332) | Non-AMD controls (n=105) | Blood donor controls (n=353) |
| --- | --- | --- | --- | --- | --- |
| Genotype |  |  |  |  |  |
| *GG* | 0.055 (n=10) | 0.040 (n=6) | 0.048 (n=16) | 0.029 (n=3) | 0.040 (n=14) |
| *CG* | 0.387 (n=70) | 0.291 (n=44) | 0.343 (n=114) | 0.248 (n=26) | 0.306 (n=108) |
| *CC* | 0.558 (n=101) | 0.669 (n=101) | 0.608 (n=202) | 0.724 (n=76) | 0.654 (n=231) |
|  |  |  |  |  |  |
| Allele |  |  |  |  |  |
| *G* | 0.249 | 0.185 | 0.220 | 0.152 | 0.193 |
| *C* | 0.751 | 0.815 | 0.780 | 0.848 | 0.807 |
