# Supplementary material for: Multifactor Effects and Evidence of Potential Interaction between Complement Factor H Y402H and LOC387715 A69S in Age-Related Macular Degeneration
Source: PLoS One. 2008 Dec 2;3(12):e3833. doi: 10.1371/journal.pone.0003833 (PMC2585793; doi:10.1371/journal.pone.0003833)
Supplement: Table S3 — (0.05 MB DOC) [file pone.0003833.s004.doc]

**Table S3.** Odds ratios **(**OR) and 95% confidence intervals (95% CI) for the Y402H polymorphism of the *complement factor H* gene, the A69S polymorphism of the *LOC387715* gene, and the R102G polymorphism of the *complement component 3* (*C3*) gene. AMD cases are compared to non-AMD controls (n=105). OR_het_ refers to a comparison of heterozygous genotype to the homozygous normal genotype, OR_hom_ to a comparison of homozygous risk genotype to the homozygous normal genotype, and OR_allele_ to comparison of risk allele to normal allele.

| Case group (n) | Risk factor | OR_het_ | 95% CI | OR_hom_ | 95% CI | OR_allele_ | 95% CI |
| --- | --- | --- | --- | --- | --- | --- | --- |
| Familial cases (181) | *CFH* | 2.18 | (1.17- 4.13) | 10.01 | (4.67-22.71) | 3.71 | (2.06-6.81) |
|  | *LOC387715* | 3.63 | (2.12-6.33) | 23.33 | (7.90-104.2) | 4.34 | (2.93-6.53) |
|  | *C3* | 2.01 | (1.18-3.51) | 2.41 | (0.70-11.62) | 1.83 | (1.18-2.90) |
|  |  |  |  |  |  |  |  |
| Sporadic cases (151) | *CFH* | 2.98 | (1.54-5.96) | 9.42 | (4.19-22.53) | 4.24 | (2.25-8.28) |
|  | *LOC387715* | 3.11 | (1.81- 5.43) | 12.11 | (3.95-55.05) | 3.14 | (2.10-4.78) |
|  | *C3* | 1.27 | (0.72-2.27) | 1.47 | (0.36-7.56) | 1.26 | (0.79-2.05) |
|  |  |  |  |  |  |  |  |
| All AMD cases (332) | *CFH* | 2.53 | (1.47-4.38) | 9.84 | (4.87-21.03) | 3.28 | (1.94-5.55) |
|  | *LOC387715* | 3.37 | (2.09-5.52) | 17.69 | (6.23-76.92) | 2.98 | (2.05-4.40) |
|  | *C3* | 1.64 | (1.00-2.75) | 1.92 | (0.61-8.82) | 1.56 | (1.04-2.41) |
