# Supplementary material for: Multifactor Effects and Evidence of Potential Interaction between Complement Factor H Y402H and LOC387715 A69S in Age-Related Macular Degeneration
Source: PLoS One. 2008 Dec 2;3(12):e3833. doi: 10.1371/journal.pone.0003833 (PMC2585793; doi:10.1371/journal.pone.0003833)
Supplement: Table S4 — (0.05 MB DOC) [file pone.0003833.s005.doc]

**Table S4.** Odds ratios **(**OR) and 95% confidence intervals (95% CI) for the Y402H polymorphism of the *complement factor H* gene, the A69S polymorphism of the *LOC387715* gene, and the R102G polymorphism of the *complement component 3* (*C3*) gene. AMD cases are compared to blood donor controls (n=350). OR_het_ refers to comparison of heterozygous genotype to homozygous normal genotype, OR_hom_ to comparison of homozygous risk genotype to homozygous normal genotype, and OR_allele_ to comparison of risk allele to normal allele.

| Case group (n) | Risk factor | OR_het_ | 95% CI | OR_hom_ | 95% CI | OR_allele_ | 95% CI |
| --- | --- | --- | --- | --- | --- | --- | --- |
| Familial cases (181) | *CFH* | 2.04 | (1.22-3.51) | 5.51 | (3.21-9.74) | 3.05 | (1.89-5.10) |
|  | *LOC387715* | 2.98 | (1.96-4.58) | 9.77 | (5.48-17.95) | 3.23 | (2.47-4.22) |
|  | *C3* | 1.48 | (1.01-2.17) | 1.64 | (0.68-3.82) | 1.39 | (1.02-1.88) |
|  |  |  |  |  |  |  |  |
| Sporadic cases (151) | *CFH* | 2.78 | (1.59-5.11) | 5.20 | (2.85-9.92) | 3.49 | (2.05-6.26) |
|  | *LOC387715* | 2.55 | (1.68-3.93) | 5.08 | (2.68-9.74) | 2.34 | (1.76-3.11) |
|  | *C3* | 0.93 | (0.61-1.42) | 0.99 | (0.34-2.58) | 0.96 | (0.67-1.34) |
|  |  |  |  |  |  |  |  |
| All AMD cases (332) | *CFH* | 2.36 | (1.55-3.64) | 5.40 | (3.42-8.66) | 3.25 | (2.19-4.89) |
|  | *LOC387715* | 2.77 | (1.98- 3.89) | 7.39 | (4.42-12.77) | 2.79 | (2.22-3.51) |
|  | *C3* | 1.21 | (0.87-1.67) | 1.30 | (0.62-2.79) | 1.18 | (0.91-1.54) |
