# Supplementary material for: Multifactor Effects and Evidence of Potential Interaction between Complement Factor H Y402H and LOC387715 A69S in Age-Related Macular Degeneration
Source: PLoS One. 2008 Dec 2;3(12):e3833. doi: 10.1371/journal.pone.0003833 (PMC2585793; doi:10.1371/journal.pone.0003833)
Supplement: Table S5 — (0.04 MB DOC) [file pone.0003833.s006.doc]

**Table S5.** Results of mutual information statistics (p<0.0167 significant).

|  | CFH Y402H | LOC387715 A69S | C3 R102G |
| --- | --- | --- | --- |
| LOC387715 | 1.6910 X 10^-6^ |  |  |
| C3 | 0.0012 | 0.0041 |  |
| Smoke | 8.24 X 10^-4^ | 0.14 | 0.1219 |
| Sex | 0.0056 | 1.04 X 10^-6^ | 0.011 |
